# An exploration of men’s experiences of undergoing active surveillance for favourable-risk prostate cancer: A mixed methods study protocol

**DOI:** 10.1186/s12885-016-2605-6

**Published:** 2016-08-02

**Authors:** Eimear Ruane-McAteer, Joe O’Sullivan, Sam Porter, Lionne Venderbos, Gillian Prue

**Affiliations:** 1 School of Nursing and Midwifery, Medical Biology Centre, Queen’s University, 97 Lisburn Road, Belfast, BT9 7BL Northern Ireland; 2 The Northern Ireland Cancer Centre (NICC), Belfast City Hospital, 51 Lisburn Road, Belfast, BT9 7AB Northern Ireland; 3 Department of Urology, Erasmus Medical Center, Rotterdam, The Netherlands

**Keywords:** Prostate cancer, Active surveillance, Psychological adjustment, Anxiety, Depression, Uncertainty, Mixed methods, Quality of life

## Abstract

**Background:**

Prostate cancer is one of the most common male cancers worldwide. Active Surveillance (AS) has been developed to allow men with lower risk disease to postpone or avoid the adverse side effects associated with curative treatments until the disease progresses. Despite the medical benefits of AS, it is reported that living with untreated cancer can create a significant emotional burden for patients.

**Methods/design:**

The aim of this study is to gain insight into the experiences of men eligible to undergo AS for favourable-risk PCa.

This study has a mixed-methods sequential explanatory design consisting of two phases: quantitative followed by qualitative. Phase 1 has a multiple point, prospective, longitudinal exploratory design. Ninety men diagnosed with favourable-risk prostate cancer will be assessed immediately post-diagnosis (baseline) and followed over a period of 12 months, in intervals of 3 month. Ninety age-matched men with no cancer diagnosis will also be recruited using peer nomination and followed up in the same 3 month intervals. Following completion of Phase 1, 10–15 AS participants who have reported both the best and worst psychological functioning will be invited to participate in semi-structured qualitative interviews. Phase 2 will facilitate further exploration of the quantitative results and obtain a richer understanding of participants’ personal interpretations of their illness and psychological wellbeing.

**Discussion:**

To our knowledge, this is the first study to utilise early baseline measures; include a healthy comparison group; calculate sample size through power calculations; and use a mixed methods approach to gain a deeper more holistic insight into the experiences of men diagnosed with favourable-risk prostate cancer.

## Background

Prostate cancer (PCa) is one of the most common male cancers worldwide [1]. This is partially attributable to the increasing use of prostate specific antigen (PSA) testing which has led to over-diagnosis, and as a result, possible overtreatment of lower risk PCa [2]. Curative treatments e.g. radical prostatectomy and radiation therapy result in substantial adverse side effects to the patient in terms of urinary, bowel and sexual dysfunction. Active surveillance (AS) has been developed in response to this in order to avoid these adverse side effects of curative treatments until the cancer progresses further. This usually consists of regular PSA tests, Digital Rectal Examinations (DRE) and annual/biannual biopsies. From a medical perspective, the efficacy of AS has been well documented [3]. However, the perception of living with untreated cancer can be an additional emotional burden for AS patients [4]. In a number of qualitative studies, men illustrated this burden by describing feelings of being uncertain, afraid and worried [5], and a perception of “risking one’s life” by undergoing AS [6].

Previous systematic reviews [7, 8] have reported that men on AS demonstrate favourable psychological wellbeing, despite the high quality of the reviews themselves the methodological limitations of the included studies suggest the need for further research. The most pertinent of these limitations included:

- Late baseline measurement, taken when participants are already on an AS protocol, reducing the ability to identify possibly significant differences in the psychological profile of those selecting AS compared to those choosing curative treatments, or to healthy peers.
- Selection of only those who have undergone curative treatments as comparators, thus allowing for comparison between the psychological effects of AS and curative interventions, however this does not enable measurement of the specific psychological effects of AS on men that could be obtained through comparison with their healthy peers. Comparison between peers with no cancer and men undergoing AS would facilitate understanding of the impact of screening, biopsy receipt, diagnosis of PCa, as well as the natural decline associated with the ageing process.
- Lack of power calculation, making it difficult to detect the significance of results;
- Use of uni-methodological approaches, which do not facilitate the combination of general and in-depth findings that can be attained by the adoption of a mixed-methods approach.

### Aims and Objectives

The overall aim of the study is to explore the experiences of men diagnosed with favourable risk prostate cancer. This will be achieved through the following objectives:

#### Primary objectives

- To determine the difference in anxiety levels between men undergoing AS for PCa, men eligible for AS yet opted for curative treatment, and age-matched non-cancer men.
- To explore and describe patients’ personal experiences of AS as a management option for favourable-risk PCa.

#### Secondary objectives

- To determine the prevalence of general anxiety, PCa specific anxiety, depression, uncertainty, and physical symptoms among men on AS.
- To compare AS patients’ depression scores and physical symptoms with men receiving curative treatments despite eligibility for AS, and age-matched non-cancer men.
- To identify the temporal variability of generic anxiety, PCa specific anxiety, depression and uncertainty in men diagnosed with favourable-risk PCa.
- To identify potential personality and/or socio-demographic characteristics predictive of patients’ decision to undergo AS over active treatment and how these characteristics predict resulting psychological and physical wellbeing.
- To explore potential differences in the experiences of men who report adverse psychological adjustment following a period of time on AS differ from those men who report favourable psychological adjustment.

## Methods/design

### Study design

A mixed-methods sequential explanatory design consisting of two phases: quantitative followed by qualitative [9], will be utilised in the proposed study. Following collection of quantitative data i.e. Phase 1, the qualitative component will be emergent from the findings of Phase 1. The results of Phase 2 will aid the interpretation of Phase 1 by providing a context within which the quantitative data can be understood. This method has been reported to be most appropriate for research which seeks to investigate relationships or trends in quantitative data while also seeking to explain the mechanism behind those trends using qualitative methods [10, 11].

Men with a history of prostate cancer and men in a similar age bracket with no diagnosis of prostate cancer have been consulted in the design of this study, including questionnaire and information sheet design, to ensure issues such as language and approach to the research is perceived to be appropriate and acceptable.

### Participants

#### Sample size

A power calculation was conducted using GPower software [12]. The sample size was calculated based on mean score and standard deviation on the State-Trait Anxiety Inventory short form (STAI-6) [13] in two populations; men on active surveillance [14] and men of a similar age group from the general population [15]. At 0.8 power, *p* value 0.05, and an effect size of 0.392 (calculated using the AS and general population data previously cited), the required sample size is 82 participants per group, 10 % was added to this to allow for attrition, resulting in a final sample size of 90 prostate cancer patients and 90 non-cancer men.

### Prostate cancer patients

#### Inclusion criteria

- Diagnosed with ‘favourable risk PCa’ i.e. low to intermediate risk PCa (Gleason score ≤7, PSA <20 ng/mL, clinical stage T1-T2b) [16]
- Eligible to undergo AS but have not yet made their treatment decision
- Deemed suitable for participation by the consultant
- Adequate understanding of the English language or a language that has validated translations of each of the scales used in the study (Phase 1). Phase 2 requires adequate understanding of English language.

#### Exclusion criteria

- Previous cancer diagnosis
- PCa that has been reclassified and is no longer eligible for AS
- Co-morbidities that would impact psychological functioning e.g. cognitive impairment
- Current or previous psychiatric diagnosis

### Age-matched non-cancer men

#### Inclusion criteria

- Male
- Aged within 5 years of age of the corresponding AS patient
- Adequate understanding of the English language or a language that has validated translations of each of the scales used in the study

#### Exclusion criteria

- Informal or formal caregiver of the corresponding AS patient
- Current or previous cancer diagnosis
- Current or previous psychiatric diagnosis
- Diagnosis that would impact psychological functioning e.g. cognitive impairment.

## Procedure

### Identification of participants

#### Prostate cancer patients

Patients diagnosed with favourable risk PCa (Gleason score ≤7, PSA <20 ng/mL and clinical stage T1-T2b) [16] who are eligible for AS will be recruited from the Northern Ireland Cancer Centre (NICC) and Belfast City Hospital (BCH). Potential participants will be identified at the regional Uro-Oncology Multi-disciplinary Team (MDT) Meeting by oncology and urology consultants. Eligible patients who present for their diagnosis/treatment decision appointment will be informed of the study by their consultant and with their permission, details passed to the research team. Potential participants will be sent an information pack including a participant information sheet and consent form, and written informed consent will be obtained prior to participation. Process consent will be used throughout the study.

#### Age-matched non-cancer men

The matched non-cancer men will be recruited using peer-nomination [17]; each participant that opts for AS will be asked to nominate a male family member or friend who meets appropriate inclusion and exclusion criteria. Peer-nomination primarily matches participants based on age, however previous studies have demonstrated that this method of recruitment also matches participants on other demographic factors e.g. education, relationship status [18, 19]. Should a participant be unable or unwilling to nominate someone, potential participants will be approached in the university and via researchers’ social circle who will then be matched to the AS patient as closely as possible in terms of their demographic profile [19].

Upon nomination of a suitable peer, the researcher will contact those nominated via telephone pending the permission of the potential non-cancer participant. Potential non-cancer participants will be sent an invitation pack which will include consent forms, information sheet and stamped addressed envelope for the return of consent form and questionnaire.

### Phase 1—Quantitative

#### Outcome measures

Demographic information will include: age; marital status; relationship status; sexual orientation; education level; employment status; ethnicity; co-morbidities (physical or psychological); other major life events in addition to the PCa diagnosis, sexual activity and personality as measured by the Eysenck Personality Questionnaire (EPQ) [20]. To assess psychological and physical functioning, the Centre for Epidemiologic Studies Depression Scale (CES-D) [21]; State-Trait Anxiety Inventory (STAI-6) [11]; Memorial Anxiety Scale for Prostate Cancer (MAX-PC) [22]; Mishel Uncertainty in Illness Scale—Community version (MUIS-C) [23]; Decisional Regret Scale [24]; EuroQol (EQ-5D-5 L) [25] and a modified version of the Expanded Prostate Cancer Index Composite (EPIC) [26] will be used.

A number of the scales to be used in the proposed study (EPQ, STAI-6, CES-D, MAX-PC) are based on a previous AS study conducted in the Netherlands [14] and therefore are deemed to be suitable for use in the target population. In addition we will assess prostate specific function (EPIC), general quality of life (EQ-5D-5 L), illness uncertainty (MUIS-C) and decisional regret (DRS). With the exception of the Decisional Conflict Scale (DCS), the scales used in the Dutch study have reported acceptable psychometric properties [13, 14, 21, 27, 28] and have been used in both prostate cancer populations and the general population previously [14, 15]. The additional scales included in the present study have also demonstrated adequate psychometric properties in the target populations [24, 29–35].

#### Prostate cancer patients (AS and AT)

**Fig. 1:**
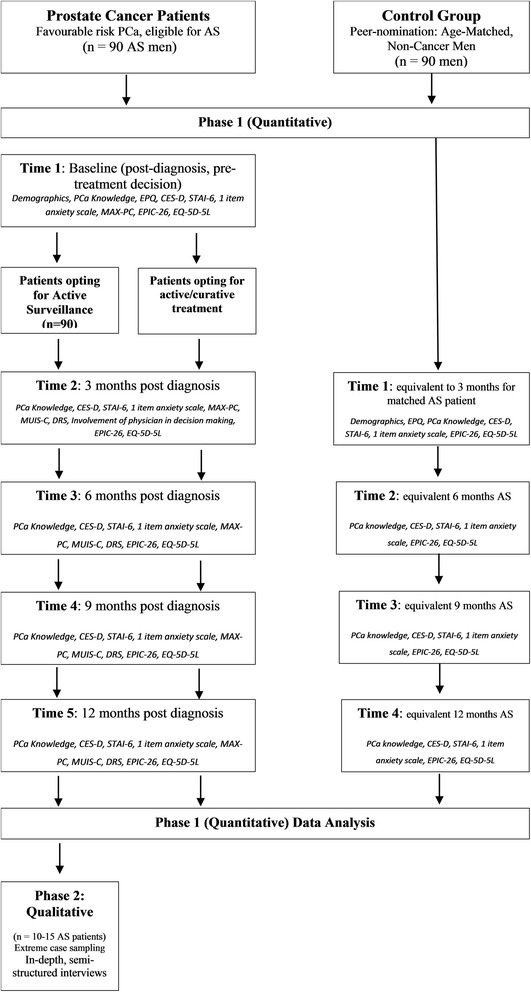
Flow diagram illustrating study procedures

At baseline, (when participants have not yet decided on their treatment approach), demographic information, depression (CES-D), anxiety (STAI-6 and MAX-PC), prostate symptoms (EPIC) and general physical health (EQ-5D-5 L) will be assessed. Three months post-commencement of treatment/AS, patients will be asked to complete CES-D, STAI-6, MAX-PC, MUIS-C, Decisional Regret Scale, EPIC, EQ-5D-5 L, items assessing PCa knowledge and involvement of the physician in decision making. The same combination of questionnaires will be completed in 3-month intervals for up to 12 months with the exception of the involvement of the physician in decision making, pending continued process consent (see Fig. 1).

To detect potential selection bias, patients who opt not to participate and those who choose to drop out of follow-up will be asked to complete a one-item anxiety Likert scale (“Please indicate the number that shows how anxious you feel at the moment.”) [36]. This one-item scale was chosen due to its high correlation with the STAI-6 and other anxiety scales [37].

#### Age-matched non-cancer men

As can be seen in Fig. 1, non-cancer participants will be assessed in 3 month intervals up to 12 months, coinciding with their corresponding AS patient’s follow-up time, using CES-D, STAI-6, EPIC and EQ-5D-5 L. At initial assessment, i.e. T3 months, participants will also be asked to report demographic information and complete both the EPQ and PCa knowledge questionnaire. Selection bias will also be assessed using the one-item anxiety scale [36].

### Phase 2—Qualitative

Following completion of Phase 1, 10–15 of those AS participants who have reported both the best and worst psychological functioning will be invited to participate in face-to-face in-depth semi-structured qualitative interviews (Fig. 1). Although participants will have provided written consent at study inception, potential Phase 2 participants will be informed of what is involved in the interviews, given an opportunity to ask questions/discuss any concerns and will be made aware of their right to refuse participation or withdraw at any point without providing an explanation.

The purpose of Phase 2 is to further explore the quantitative results of Phase 1, to gain a richer understanding of participants own interpretation of their illness and its impact on their psychological wellbeing [38], be it positive or negative. A recent systematic literature review conducted by the research team, along with the outcome of Phase 1, will be used to frame topics for the semi-structured interviews [10], however participants will also be encouraged to discuss issues that they feel are of importance to them. Transcripts of each interview will be audio recorded, and transcribed verbatim.

Participants will be asked to suggest an interview location where they feel most comfortable which may include their own homes or the designated research room in the cancer centres. Interviews are expected to last for approximately 60 min however this is dependent on the participants’ willingness to talk and the depth they wish to discuss the topics.

Although interviews will consist of a semi-structured format, with a range of predetermined topics, participants will be encouraged to discuss issues of personal importance that the quantitative phase or the phase 2 topic guide may not have addressed. Participants will be given the opportunity to articulate their own personal and unique experiences of PCa and AS. Participants will be viewed as ‘experiential experts’ while exploring their interpretations of their experiences of prostate cancer and active surveillance. Verbal and non-verbal observations will be recorded immediately post-interview in a field diary to document subtleties that may not be picked up via audio recorder e.g. mood, emotion, body language. This field diary will be used to aid data interpretation and analysis [39].

Participants may struggle to discuss issues, such as sexual symptoms or psychological distress, with the researcher. To overcome this, the researcher will place emphasis on developing rapport with the participant, assuming an open and non-judgemental approach and ensuring the participant is aware of the strict confidence within which their information will be kept. Previous research has shown that men with prostate cancer embrace the opportunity to discuss personal issues in a confidential research setting outside of their immediate social circle and medical team, and are willing to provide rich data on the topic [40].

## Analysis

### Phase 1—Quantitative

Data analysis will be performed using the Statistical Package for the Social Sciences (SPSS) Version 22 for Windows [41].

Basic data will be collected on all men screened. Care will be taken to ensure the individual’s anonymity is protected. The number of men screened, those eligible and ineligible, those accrued and those not willing to participate with reasons for ineligibility and non-participation will be recorded and documented. This data will be examined and descriptive analysis carried out to identify any differences between participants and non-participants.

Demographics will be summarised using descriptive statistics for AS, AT and non-cancer participants. AS and AT participants’ clinical data will also be analysed using descriptive statistics.

To detect selection bias, a univariate analysis (*t*-test) will be used to compare the difference in the scores of a one-item anxiety Likert scale [36] of those who agreed to participate and those who did not agree or withdrew from the study.

Hierarchical linear modelling (HLM) will be used to analyse the data collected. Use of this method, presents an opportunity to explore the differences, as well as the demographic and clinical determinants, in psychological wellbeing at a group level (i.e. AS, AT, Non-Ca), and an individual level. HLM further facilitates the exploration of temporal variability and determinants of this variability and patterns of change across individual patients and participant groups [42]. In longitudinal study designs, attrition and missing data is a concern and can limit the power and interpretability of the study, HLM addresses this issue in its ability to create the model in spite of missing or incomplete data [43].

Due to the increased ability to separate individual and group level variation using HLM, this approach is in keeping with the rationale of this study in terms of maintaining a patient-centred approach [43], therefore allowing the identification of the wider experience of men undergoing AS, relative to the other included groups under study, while also remaining sensitive to the variation in individual experience of AS and PCa.

### Phase 2—Qualitative

Transcriptions of audio-recorded interviews will be analysed using thematic analysis [44].

Thematic analysis is a “method of identifying, analysing and reporting pattern themes within data” [44]. Analysis will be performed in three stages: data reduction, data display and conclusion drawing/verification process [45]. At each stage, findings will be discussed and verified by the research team in order to assess accuracy of the interpretation, promote inter-rater reliability and ensure rigour in the qualitative phase of the research [46].

NVivo qualitative data analysis software version 10.0 [47] will be used to aid data management.

## Integration of Findings

Findings from qualitative and quantitative phases will be integrated at the analysis stage. Each phase will be analysed separately and summarised in a mixed methods matrix in order to integrate the two sets of results [48]. A mixed methods matrix allows researchers to present a summary of both quantitative and qualitative data together, which draws attention to patterns, surprises and paradoxes in single cases and across the sample as a whole. This technique is increasingly being utilised in healthcare research due to the added insight it provides by analysing data obtained from both methodological approaches together [48].

### Ethical considerations

The recruitment of men at the point where they find out they have prostate cancer presents a potential ethical issue relating to their psychological vulnerability at this time. However, these men will only be approached with the view to provide them with the study information pack and to obtain permission to contact them via telephone 7–14 days later in order to discuss the study. Men will not be asked to provide consent or to complete the questionnaire at this point.

In terms of recruiting non-cancer participants, it is possible that participating in this study may motivate them to attend screening for PCa and potentially be diagnosed with PCa. This poses an ethical issue, however due to the use of peer-nomination as the recruitment strategy, it is anticipated that this will minimise this issue due to the control participants’ already heightened awareness of PCa due to the diagnosis of a close family member or friend. In any case, should a control participant receive a diagnosis of PCa during the course of follow-up, no further data will be collected from them; data received prior to diagnosis will be retained in the dataset.

The autonomy of participants is central to this investigation. Participants will be made aware that consent is fluid and that they have the right to withdraw their consent at any time throughout the study without any negative impact on their healthcare or management. The researcher will also adhere to the principle of non-maleficence, above all cause no harm to participants.

All potential participants will have all of the necessary information for informed consent and had the opportunity to ask questions about the study. Written consent will be obtained without any coercion of study participants. Confidentiality will be assured and the Caldicott Principles adhered to. Participants will be made aware that any digital recordings will be deleted following transcription. All information pertaining to individuals will be anonymised from the outset of the study and consent sought for the use of anonymised quotations from participants.

Data will be protected under the provisions of the Data Protection Act 1998 [49] – the data will only be used for the purpose of the study, no participant will be identifiable in any way and all data will be stored in a locked container in a locked office. Information stored electronically will be stored on a password-protected computer for 3 years in line with university policy. Should electronic information need to be transferred, password-protected encrypted pen-drives will be used.

In line with the guidance that normally only a member of the patient’s existing clinical care team should have access to patient records, the consultant/clinical nurse specialist will identify potential participants. To ensure no patient is approached unnecessarily, the researcher will be present at the clinic to clarify any uncertainties (anonymously) with the consultant/clinical nurse specialist.

### Dissemination

Following completion of the study, findings will be written up for submission to peer-reviewed journals and conference presentations. Summary of findings will be prepared for consultants and nurse specialists involved in the study. A lay summary will also be prepared for dissemination to study participants.

## Discussion

The present study has been designed to capture a baseline assessment of men’s psychological wellbeing before they make their treatment decision, therefore conclusions can be made regarding the potential bias in terms of the type of patient that opt for AS or AT. Due to the longitudinal nature of the study, the experiences of men at various points in their first 12 months of the cancer journey can be better understood, particularly if there are specific times at which additional support is required. Comparison with men who have no diagnosis of cancer and those who opt for AT is a further strength in terms of placing the data in context to support the analysis and interpretation of results. Use of a power calculation in order to determine the appropriate sample size and inclusion of the one item anxiety question for non-responders and those who wish to be excluded from follow-up will increase validity and robustness of the findings. Finally, the collection of both qualitative and quantitative data will facilitate a more holistic understanding of men’s experiences of favourable-risk PCa.

To our knowledge, this study will be the first to include an early baseline measurement taken before treatment modalities have been decided; adopt a control group of men not diagnosed with cancer; use power calculations to ascertain required sample size; and combine qualitative and quantitative methodologies. The purpose of adopting these innovative methodological strategies is to attain a more complete picture of these men’s experiences.

## Abbreviations

AS, active surveillance; AT, active treatment; BCH, Belfast City Hospital; CES-D, Centre for Epidemiological Studies Depression scale; DCS, Decisional Conflict Scale; DRE, Digital Rectal Examination; EPIC, Expanded Prostate Cancer Index Composite; EPQ, Eysenck Personality Questionnaire; EQ-5D-5 L, EuroQOL questionnaire - five dimensions – five response levels; MAX-PC, Memorial Anxiety Scale for Prostate Cancer; MBC, Medical Biology Centre; MDT, Multi-disciplinary team; MUIS-C, Mishel Uncertainty in Illness Scale – Community Form; NICC, Northern Ireland Cancer Centre; PCa, Prostate Cancer; PSA, Prostate specific antigen; QUB, Queen’s University Belfast; RMM, Repeated measurements modelling; SF-12, Short Form – 12; SPSS, Statistical Package for the Social Sciences; STAI, State Trait Anxiety Inventory

## References

[1] Ferlay J, Soerjomataram I, Ervik M, Dikshit R, Eser S, Mathers C, Rebelo M, Parkin DM, Forman D, Bray F. Cancer incidence and mortality worldwide: sources, methods and major patterns in GLOBOCAN 2012. Int J Cancer. 2013; doi:10.1002/ijc.29210. 10.1002/ijc.29210 25220842

[2] Lawrentschuk N, Klotz L (2010). Active surveillance for favorable-risk prostate cancer: A short review. Korean J Urol.

[3] Wilt TJ, Brawer MK, Jones KM, Barry MJ, Aronson WJ, Fox S, Wheeler T (2012). Radical prostatectomy versus observation for localized prostate cancer. N Eng J Med.

[4] Bailey DE, Wallace M, Mishel MH (2007). Watching, waiting and uncertainty in prostate cancer. J Clin Nurs.

[5] Hedestig O, Sandman P-O, Widmark A (2003). Living with untreated localized prostate cancer. Cancer Nurs.

[6] Kazer MW (2012). Conversion from active surveillance to active treatment for prostate cancer: A qualitative analysis. J Nurs Educ Pract.

[7] Bellardita L, Valdagni R, van den Bergh R, Randsdorp H, Repetto C, Venderbos LDF, … Korfage IJ. How Does Active Surveillance for Prostate Cancer Affect Quality of Life? A Systematic Review. Eur Urol. 2014; 1–9. doi:10.1016/j.eururo.2014.10.028. 10.1016/j.eururo.2014.10.028 25454617

[8] Carter G, Clover K, Britton B, Mitchell AJ, White M, McLeod N, Denham J, Lambert SD (2015). Wellbeing during Active Surveillance for localised prostate cancer: A systematic review of psychological morbidity and quality of life. Cancer Treat Rev.

[9] Creswell JW, Plano Clark VL, Gutmann ML, Hanson WE (2003). Advanced mixed methods research designs. Handbook of mixed methods in social and behavioral research.

[10] Creswell JW, Plano Clark VL (2007). Designing and conducting mixed methods research.

[11] Tashakkori A, Teddlie C (1998). Mixed methodology: Combining qualitative and quantitative approaches.

[12] Faul F, Erdfelder E, Lang AG, Buchner A (2007). G* Power 3: A flexible statistical power analysis program for the social, behavioral, and biomedical sciences. Behav Res Methods.

[13] Marteau TM, Bekker H (1992). The development of a six-item short-form of the state scale of the Spielberger State-Trait Anxiety Inventory (STAI). Br J Clin Psychol.

[14] van den Bergh RCN, Essink-Bot M-L, Roobol MJ, Wolters T, Schröder FH, Bangma CH, Steyerberg EW. Anxiety and distress during active surveillance for early prostate cancer. Cancer. 2009; doi:10.1002/cncr.24446. 10.1002/cncr.24446 19637245

[15] Venderbos LDF, Korfage IJ, Roobol MJ (2015). Quality of life of men on active surveillance for prostate cancer versus men without prostate cancer: are there any differences?. Eur Urol Supplement.

[16] National Institute for Health and Clinical Excellence. Prostate Cancer: diagnosis and treatment (CG175). 2014. http://www.nice.org.uk/guidance/CG175. Accessed 1 Aug 2016.

[17] Jacobsen PB, Hann DM, Azzarello LM, Horton J, Balducci L, Lyman GH (1999). Fatigue in women receiving adjuvant chemotherapy for breast cancer: characteristics, course, and correlates. J Pain Symptom Manage.

[18] Logan HL, Tomar SL, Chang M, Turner GE, Mendenhall WM, Riggs CE (2012). Selecting a comparison group for 5-year oral and pharyngeal cancer survivors: Two methods. BMC Med Res Methodol.

[19] Prue G, Allen J, Gracey J, Rankin J, Cramp F (2010). Fatigue in gynecological cancer patients during and after anticancer treatment. J Pain Symptom Manage.

[20] Eysenck HJ, Eysenck SBG (1991). Manual of the Eysenck Personality Scales (EPS Adult).

[21] Radloff LS (1977). The CES-D scale: A self report depression scale for research in the general population. Appl Psychol Meas.

[22] Roth AJ, Rosenfeld B, Kornblith AB, Gibson C, Scher HI, Curley‐Smart T, Breitbart W (2003). The memorial anxiety scale for prostate cancer. Cancer.

[23] Mishel MH (1997). Uncertainty in Illness Scales manual. Available from M Mishel at the University of North Carolina-Chapel Hill.

[24] O’Connor AM (1996). User manual – decision regret scale [document on the Internet].

[25] The EuroQol Group (1990). EuroQol-a new facility for the measurement of health-related quality of life. Health Policy.

[26] Wei J, Dunn R, Litwin M, Sandler H, Sanda M (2000). Development and Validation of the Expanded Prostate Cancer Index Composite (EPIC) for comprehensive assessment of health-related quality of life in men with prostate cancer. Urology.

[27] Sato T (2005). The eysenck personality questionnaire brief version: factor structure and reliability. J Psychol.

[28] Roth A, Nelson CJ, Rosenfeld B, Warshowski A, O’Shea N, Scher H, Breitbart W (2006). Assessing anxiety in men with prostate cancer: further data on the reliability and validity of the Memorial Anxiety Scale for Prostate Cancer (MAX–PC). Psychosomatics.

[29] Brehaut JC, O’Connor AM, Wood TJ, Hack TF, Siminoff L, Gordon E, Feldman-Stewart D (2003). Validation of a decision regret scale. Med Decis Making.

[30] Szymanski KM, Wei JT, Dunn RL, Sanda MG (2010). Development and validation of an abbreviated version of the expanded prostate cancer index composite instrument for measuring health-related quality of life among prostate cancer survivors. Urology.

[31] Bailey DE, Wallace M, Latini DM, Hegarty J, Carroll PR, Klein EA, Albertsen PC (2011). Measuring illness uncertainty in men undergoing active surveillance for prostate cancer. Appl Nurs Res.

[32] Mishel M, Epstein D (1990). Uncertainty in illness scales: manual.

[33] Janssen MF, Birnie E, Haagsma JA, Bonsel GJ (2008). Comparing the standard EQ-5D three level system with a five-level version. Value Health.

[34] Janssen MF, Pickard AS, Golicki D, Gudex C, Niewada M, Scalone L, Busschbach J (2013). Measurement properties of the EQ-5D-5 L compared to the EQ-5D-3 L across eight patient groups: a multi-country study. Qual Life Res.

[35] Patient Reported Outcome Measurement Group Oxford (2009). A structured review of patient reported outcome measures (PROMs) for prostate cancer. Report to the Dept of health.

[36] Davey H, Barratt AL, Butow PN, Deeks JJ (2007). A one-item question with a Likert or Visual Analog Scale adequately measured current anxiety. J Clin Epidemiol.

[37] Bindhim NF, Shaman AM, Alhawassi TM (2013). Confirming the one-item question likert scale to measure anxiety. Internet J Epidemiol.

[38] Creswell JW (2013). Research design: Qualitative, quantitative, and mixed methods approaches.

[39] Goodwin D (2006). Ethical issues in qualitative research in health care.

[40] McSorley O, McCaughan E, Prue G, Bunting B, Parahoo K, O’Sullivan J (2013). A longitudinal study of coping strategies in men receiving radiotherapy and neo-adjuvant androgen deprivation for prostate cancer: a quantitative and qualitative study. J Adv Nurs.

[41] Corp IBM (2013). IBM SPSS statistics for windows, version 22.0.

[42] Raudenbush SW, Bryk AS (2002). Hierarchical linear models: applications and data analysis methods.

[43] Lininger M, Spybrook J, Cheatham CC (2015). Hierarchical linear model: thinking outside the traditional repeated-measures analysis-of-variance box. J Athl Train.

[44] Braun V, Clarke V (2006). Using thematic analysis in psychology. Qual Res Psychol.

[45] Miles MB, Huberman AM (1994). Qualitative data analysis: An expanded sourcebook.

[46] Mays N, Pope C (1995). Rigour and qualitative research. BMJ.

[47] Richards L (1999). Using NVivo in Qualitative Research.

[48] O’Cathain A, Murphy E, Nicholl J (2010). Three techniques for integrating data in mixed methods studies. BMJ.

[49] Data Protection Act 1998. www.uk-legislation.hmso.gov.uk/acts/acts1998/19980029.html. (Accessed 1 Aug 2016).

